# Modeling Single Nucleotide Polymorphisms in the Human *AKR1C1* and *AKR1C2* Genes: Implications for Functional and Genotyping Analyses

**DOI:** 10.1371/journal.pone.0015604

**Published:** 2010-12-31

**Authors:** Jonathan W. Arthur, Juergen K. V. Reichardt

**Affiliations:** 1 Discipline of Medicine, Sydney Medical School, University of Sydney, Sydney, New South Wales, Australia; 2 Plunkett Chair of Molecular Biology (Medicine), Sydney Medical School, University of Sydney, Sydney, New South Wales, Australia; Ohio State University Medical Center, United States of America

## Abstract

Enzymes encoded by the AKR1C1 and AKR1C2 genes are responsible for the metabolism of progesterone and 5α-dihydrotestosterone (DHT), respectively. The effect of amino acid substitutions, resulting from single nucleotide polymorphisms (SNPs) in the AKR1C2 gene, on the enzyme kinetics of the AKR1C2 gene product were determined experimentally by Takashi *et al*. In this paper, we used homology modeling to predict and analyze the structure of AKR1C1 and AKR1C2 genetic variants. The experimental reduction in enzyme activity in the AKR1C2 variants F46Y and L172Q, as determined by Takahashi *et al.*, is predicted to be due to increased instability in cofactor binding, caused by disruptions to the hydrogen bonds between NADP and AKR1C2, resulting from the insertion of polar residues into largely non-polar environments near the site of cofactor binding. Other AKR1C2 variants were shown to involve either conservative substitutions or changes taking place on the surface of the molecule and distant from the active site, confirming the experimental finding of Takahashi *et al.* that these variants do not result in any statistically significant reduction in enzyme activity. The AKR1C1 R258C variant is predicted to have no effect on enzyme activity for similar reasons. Thus, we provide further insight into the molecular mechanism of the enzyme kinetics of these proteins. Our data also highlight previously reported difficulties with online databases.

## Introduction

**Figure 1:**
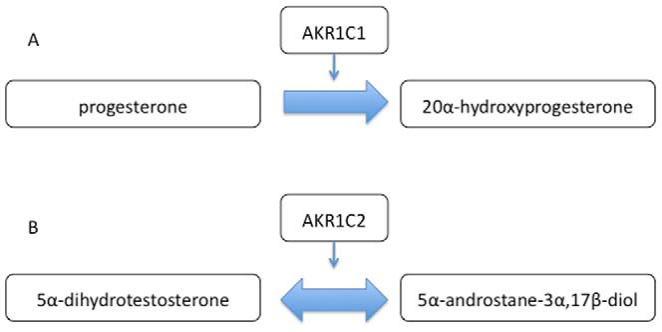
Reactions catalyzed by the AKR1C1 and AKR1C2 gene products. The AKR1C1 gene product catalyzes the transformation of progesterone into 20α-hydroxyprogesterone (Panel A). The AKR1C2 gene product stereospecifically metabolizes 5α-dihydrotestosterone (DHT) to 5α-androstane-3α,17β-diol (Panel B).

The aldo-keto reductase superfamily includes a variety of oxidoreductases with a common dependence on NADPH for their enzyme activity[1]. In particular, the AKR1C1 and AKR1C2 genes encode two 3α-hydroxysteroid dehydrogenase isoforms of the aldo-keto reductase superfamily responsible for the inactivation and formation of male and female sex hormones[2]. The enzyme encoded by the AKR1C1 gene catalyzes the transformation of progesterone into 20α-hydroxyprogesterone and thus plays a critical role in controlling plasma progesterone levels during pregnancy[3] (**Figure 1**). The AKR1C2 gene product stereospecifically metabolizes 5α-dihydrotestosterone (DHT) to 5α-androstane-3α,17β-diol and thus plays a critical role in regulating androgen receptor signaling in the prostate[4] (**Figure 1**). DHT is a key molecule in prostate cancer development and alterations in the expression of AKR1C2 have been linked to benign prostatic hyperplasia and prostate cancer[5], [6].

Takahashi *et al.* [4] reported the effect of eleven amino acid substitutions encoded by the AKR1C2 gene on the activity of the gene product *in vitro*. Using the list of non-synonymous single-nucleotide polymorphisms (SNPs) provided by the AKR website (www.med.upenn.edu/akr), in turn obtained from the dbSNP database[7] at the National Center for Biotechnology Information (NCBI), they created expression constructs for the wild-type (normal) and eleven variants. Subsequently, they performed a kinetic analysis to measure both enzyme activity (measured as *V_max_*) and substrate binding (measured as *K_m_*) in the reduction of DHT. A significant reduction in enzyme activity was seen in the F46Y and L172Q variants and a significant increase in substrate binding was seen in V38A, L172Q, K185E, and R258C[4].

However, not all of the constructs tested correspond to natural sequence variants (known as single nucleotide polymorphisms or SNPs). We have previously shown that as many as 8.3% of the single nucleotide polymorphisms (SNPs) recorded in the dbSNP are actually single nucleotide differences (SNDs)[8], [9]. That is, they are not SNPs but are artifacts arising from the presence of a paralogue (*i.e.*, highly similar duplicated) sequence in the genome. In fact, six variants examined by Takashi *et al.* [4] appear to be such artefactual SNDs.

In this paper, we used homology modeling to predict the structure of the various AKR1C1 and AKR1C2 isoforms derived from either the actual non-synonymous SNPs in the dbSNP databases or the AKR1C2 SNDs. By analyzing the local structural environment around the substituted amino acids, we were able to develop physiochemical hypotheses as to the effect of the substitution on enzyme activity and co-factor binding. Furthermore, we suggest further experiments that could be undertaken to confirm these findings and thus improve our understanding of the molecular basis of AKR1C1 and AKR1C2 functionality. Lastly this paper highlights again the perils associated with some online databases.

## Methods

The list of eleven non-synonymous AKR1C2 SNPs studied by Takahashi *et al.* [4] was compared to list provided on the AKR website (www.med.upenn.edu/akr) as well as the list of AKR1C2 SNPs and the database of SNDs prepared by Musumeci *et al.* [8]. Finally, all SNPs were reviewed in the current version of the dbSNP database (October 2010) to check for recent updates. All but one of the eleven (rs2518043, leading to K185E) were listed on the AKR website. All eleven were still current compared to the latest version of dbSNP, although three (rs10618, rs11474, and rs28943580) were listed as SNPs in both AKR1C2 and AKR1C1. After comparison with the SND database, only five were confirmed as legitimate SNPs (rs2854482, rs2854486, rs2518042, rs2518043, and rs28943580) while another five (rs3207898, rs3207901, rs3207905, rs13933, and rs10618) were identified as SNDs. rs11474 was not included in the SNPs tested in Musumeci *et al.* [8] but was confirmed to be a SND by using an analogous procedure.

All five SNPs and the six SNDs were subjected to homology modeling for theoretical comparison with the experimental data. **Table 1** shows the AKR1C2 SNPs and SNDs modeled, the corresponding amino acid change, and a summary of our findings along with those of Takahashi *et al*.

**Table 1:** A list of the non-synonymous AKR1C1 and AKR1C2 SNPs modeled showing the amino acid change and the predicted and actual effects on enzyme activity, substrate binding, and cofactor binding.

SNP/SND	Substitution	Effect on enzyme activity (*V_max_*)		Effect on substrate binding (*K_m_*)		Effect on cofactor binding (*K_m_*)
*SNPs*		*Predicted*	*Actual*	*Predicted*	*Actual*	*Predicted*
rs2854482	F46Y	***Decrease***	***Decrease***	***No change***	No change*	Increase
rs2854486	V111A	***No change***	No change*	***No change***	No change*	No change
rs2518042	K179E	***No change***	No change*	***No change***	No change*	No change
rs2518043	K185E	***No change***	No change*	No change	Decrease	No change
rs28943580	R258C (AKR1C2)	***No change***	No change*	No change	Decrease	No change
rs28943580	R258C (AKR1C1)	No change	-	No change	-	No change
*SNDs*						
rs3207898	V38I	***No change***	No change*	***No change***	No change*	No change
rs3207901	V38A	***No change***	No change*	No change	Decrease	No change
rs3207905	H47R	***No change***	No change*	***No change***	No change*	No change
rs13933	S87C	***No change***	No change*	***No change***	No change*	No change
rs10618	H170R	***No change***	No change*	***No change***	No change*	No change
rs11474	L172Q	***Decrease***	***Decrease***	No change	Decrease	Increase

The first five SNPs are from AKR1C2 and the last SNP is from AKR1C1. The SNDs are all from AKR1C2. Actual effects on enzyme activity and substrate binding in AKR1C2 are determined by comparing the experimental values of *V_max_* and *K_m_* for the variant with those of the WT (normal) using data from Table 3 in Takahashi *et al.* [4]. An asterisk indicates the difference measured by Takahashi *et al.* did not reach statistical significance and is thus reported here as “no change”. An increase in *K_m_* corresponds to the substrate or cofactor being more weakly bound. No experimental data is provided for AKR1C1 or cofactor binding in AKR1C2 as these were not studied. The “predicted” data summarizes our findings in this paper. Cells highlighted in bold-italic indicate where the predictions derived from our modeling agree with the experimental data.

Each variant of the AKR1C2 protein was modeled using MolIDE[10] and the following procedure. The FASTA format sequence of AKR1C2 was taken from UniProt[11] (accession number: P52895) and edited to reflect the amino acid change in the variant. The edited sequence was compared to the UniRef100 database using PSI-BLAST[12] to construct a sequence profile. The sequence profile was then used to compare the variant sequence with the Protein Data Bank (PDB)[13] to identify proteins of known structure with sequence homology to the variant. Five crystal structures of human AKR1C2 were identified with zero E-value and sequence identity of 99%. 1J96[14] was chosen as a template for homology modeling due to the presence of the testosterone substrate in the crystal structure; this being the most similar substrate in any of the potential template structures to DHT. The homology model was constructed with the position of the backbone atoms and conserved side-chains assigned according to the alignment with 1J96. The side chain for the mutated residue was built using SCWRL4[15].

The list of AKR1C1 SNPs in Musumeci *et al.* [8] were reviewed in the current version of the dbSNP database (October 2010) to check for updates. The three non-synonymous variants were still current and consistent with the information on the AKR website. All three were confirmed as SNDs using the SND database[8]. As noted above, the review of the AKR1C2 SNPs identified three SNPs also in AKR1C1. Of these, only one was a legitimate SNP: rs28943580 resulting in a R258C variant. The homology model for this variant was produced using the same method as described above. The UniProt sequence for AKR1C1 (accession number: Q04828) was edited to create the R258C variant. Two homologous AKR1C1 crystal structures were identified and 1MRQ[3] was chosen as a template due to the presence of the progesterone substrate in the crystal structure.

Homology models were visualized and molecular graphics images produced using the UCSF Chimera package from the Resource for Biocomputing, Visualization, and Informatics at the University of California, San Francisco (supported by NIH P41 RR-01081)[16].

In order to quantify the structural changes resulting from the SNPs, the wild type and the SNP variant structures were evaluated using a range of structure assessment software. The ProSA-web[17], [18] z-score of the variants and the wild types structures was used to determine any change in the quality of the structure as a result of the mutation. Verify3D[19], [20] was used to check for improperly built segments. The range of scores over the whole protein and individual scores for the mutated residues were compared with the wild type to identify any structural problems arising from the mutation. MolProbity[21], [22] clash scores for both the whole protein and individual residues were also compared to identify structural clashes resulting from the mutations. Finally, naccess (http://www.bioinf.manchester.ac.uk/naccess/naccess.html), using the method of Lee and Richards[23] was used to quantify any changes in the absolute accessible surface area of the side chain as a result of the mutations.

## Results

**Figure 2:**
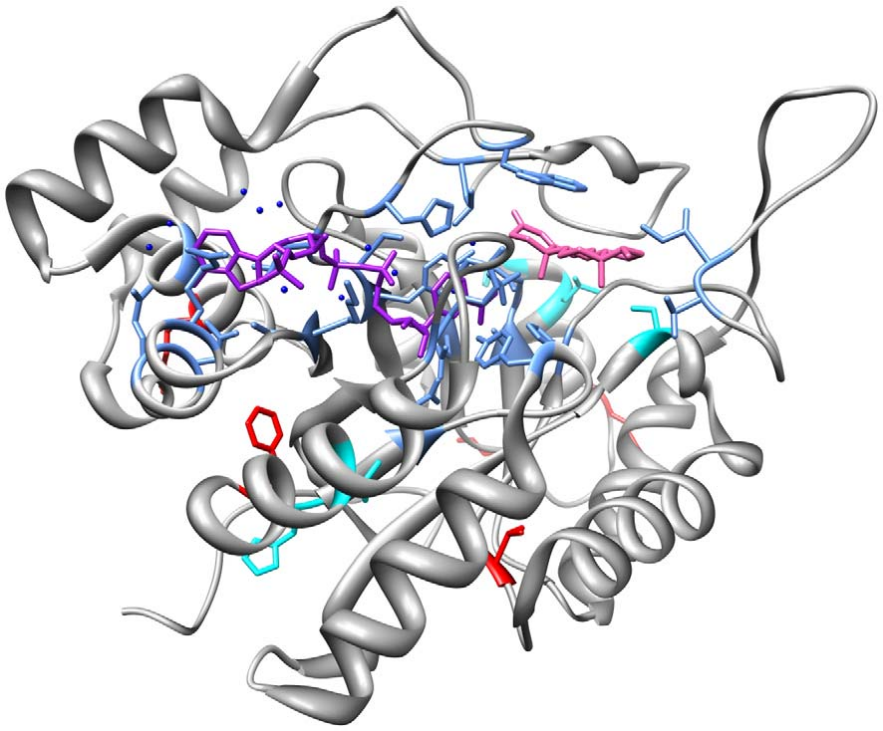
The structure of AKR1C2. The structure shows chain A from the PDB record 1J96. The substrate is shown in pink and the cofactor is shown in purple. Water molecules are shown in blue. The residues varied as a result of a SNP (F46, V111, K179, K185, and R258) are shown in red and residues varied as a result of a SND (V38, H47, S87, H170, and L172Q) are shown in cyan. Residues around the active site are shown in light blue.

The list of non-synonymous AKR1C2 SNPs, *i.e.*, those resulting in an amino acid change in the expressed protein, studied by Takahashi *et al.* [4] was compared to the database of SNDs prepared by Musumeci *et al.* [8] and the legitimate SNPs (rs2854482, rs2854486, rs2518042, rs2518043, and rs28943580) were identified. All SNPs and SNDs were subjected to homology modeling (Table 1). Each variant of the AKR1C2 protein was modeled using MolIDE[10], as per the procedures detailed under Materials and Methods, in order to obtain a three-dimensional structure for each of the variants. Lastly, these homology models were visualized and molecular graphics images produced using the UCSF Chimera package from the Resource for Biocomputing, Visualization, and Informatics at the University of California, San Francisco (supported by NIH P41 RR-01081)[16]. By visualizing the position of the substituted amino acid, the other amino acids in the immediate environment, and the proximity of the substituted amino acid to the substrate and the cofactor, it is possible to suggest a physiochemical rationale for the effect, or the lack of any effect, of the substitution on enzyme kinetics as determined by the experiments of Takahashi *et al.* [4]. **Figure 2** shows the structure of AKR1C2, indicating the position of the substrate, cofactor, active site, and variant residues. The results for each SNP or SND examined are reported individually in detail below.

### AKR1C2 SNP variants

#### F46Y variant

**Figure 3:**
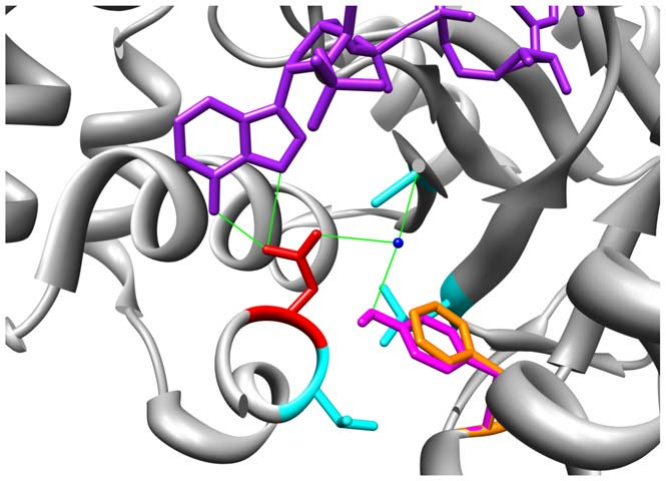
The F46Y amino acid substitution in the predicted AKR1C2 protein disrupts NADP cofactor binding. The environment of residue 46 shows both the F (orange) and Y (magenta) variants adopt the same rotamer. The introduction of the polar Y residue into an otherwise hydrophobic environment is likely to disrupt the hydrogen bonding (green) between N280 (red) and the NADP molecule (purple) and nearby water molecule (blue), thereby disrupting NADP binding. Helix residues 271–278 lie over the top of the area depicted and have been removed from the image for clarity.

The rs2854482 SNP results in the substitution of tyrosine (single letter amino acid code: Y) for phenylalanine (F) at position 46 in the protein sequence (in short F46Y). This exchanges a large hydrophobic residue for a large polar residue. **Figure 3** shows the environment around residue 46 in the predicted AKR1C2 protein. Both the F and Y residues adopt the same rotamer. The normal or wild type (WT) F46 (shown in orange; **Figure 3**) exists in a generally hydrophobic environment comprising L19, A269, and V281 (shown in cyan). The exception is N280 (shown in red), which forms hydrogen bonds with the adenine ring nitrogen atoms in the NADP cofactor[24] (shown in purple) and a water molecule (shown in blue).

Substitution of Y for F results in a slight improvement in ProSA-web z-score, from −11.63 to −11.65, while the Verify3D score is slightly worse: up to 0.67 from 0.61. The MolProbity clash score rises from 6.96 to 16.64 and identifies a potential clash between Y46 and N280 (0.489 Å): a likely hydrogen bond as noted below. There is almost no change in the accessible surface area (2.35 to 2.56).

The F46Y substitution (shown in magenta; **Figure 3**) introduces a polar residue into this largely hydrophobic environment. The oxygen of Y46 can also form a hydrogen bond with the water molecule lying between it and N280. This suggests the F46Y substitution may change the local environment in such a way as to disrupt the hydrogen bonds of N280 and thus destabilize the binding of the NADP cofactor.

This finding concurs with, and potentially explains, the previous experimental findings of Takahashi *et al.* [4], who showed a significant reduction in enzyme activity (*V_max_*) as a result of the F46Y mutation, as activity would be reduced by poorer cofactor binding. In addition, they did not find a statistically significant change in DHT binding. This also concurs with our results as the F46Y mutation is located at some distance from the testosterone substrate of the model, suggesting the reduction in enzyme activity is mediated through the changes to the interaction between the enzyme and the cofactor rather than the interaction of the former with the substrate (**Table 1**).

#### V111A variant

The V111A variant is a result of the rs2854486 SNP. It involves the conservative exchange of a small hydrophobic residue (valine) for a slightly smaller hydrophobic residue (alanine). Figure S1 shows the environment around residue 111. Residue 111 is located away from the active site of AKR1C2 and on the periphery of the molecule. The environment around residue 111 is generally hydrophobic, comprising L99, L103, L106, and A160 (shown in cyan).

The V111A substitution results in a slight worsening in ProSA-web z-score, from −11.63 to −11.51, while the Verify3D score is slightly better: down to 0.64 from 0.66. The MolProbity clash score rises from 6.96 to 16.27 but no new clash arises from the substitution. There is almost no change in the accessible surface area (0.00 to 0.37).

The conservative nature of the substitution, its distance from the active site, and its location on the periphery, suggest V111A would have no effect on enzyme activity or substrate binding. These data concur with the findings of Takahashi *et al.* [4] who showed no significant difference in either *V_max_* or *K_m_* for the V111A variant (**Table 1**).

#### K179E variant

The K179E variant is a result of the rs2518042 SNP. It involves the exchange of a large positively charged residue, lysine (K), for a smaller negatively charged residue glutamate (E). Figure S2 shows the environment around residue 179. Both K (shown in orange) and E (shown in magenta) adopt the same rotamer. The normal (WT) K179 potentially forms a salt bridge with the neighbouring E149 and E152 (shown in cyan). In contrast, the K179E variant breaks this salt bridge and, due to the smaller size of the glutamate residue, draws this negative charge further into the non-polar core of the protein, bringing it into proximity with W148, I176, and L182 (shown in cyan).

Substitution of E for K at position 179 results in a slight improvement in ProSA-web z-score, from −11.63 to −11.66 and the Verify3D score: down to 0.28 from 0.37. The MolProbity clash score rises from 6.96 to 16.08 and identifies a potential clash between E179 and the P180 backbone (0.464 Å). There is a slight increase in the accessible surface area (18.93 to 19.94).

Despite these changes, residue 179 is located in a loop region, on the surface of the protein, and distant from the active site. As such, it suggests the change is unlikely to affect either enzyme activity or substrate binding. This agrees with the findings of Takahashi *et al.* [4] who showed no significant difference in either *V_max_* or *K_m_* for the K179E variant (**Table 1**).

#### K185E variant

As for the previous variant, the rs2518043 SNP, results in the exchange of a large positively charged residue, lysine, for a smaller negatively charged residue, glutamate. Figure S3 shows the environment around residue 185. Both K and E adopt the same rotamer. The normal (WT) K185 residue (shown in orange) exists in a polar environment formed by N11, K209 (shown in cyan), and several water molecules (shown in blue). Once again, the K185E substitution (shown in magenta) disrupts these polar interactions and draws the negative charge further into the non-polar core of the protein, bringing it into proximity with L177 and L182 (shown in cyan).

The K185E substitution results in no change to the ProSA-web z-score of −11.63 or the Verify3D score of 0.20. The MolProbity clash score rises from 6.96 to 16.28 but no new clash arises from the substitution. The accessible surface area is slightly reduced (65.99 to 62.70).

Residue 185 is also located in a loop region, on the surface of the protein, and distant from the active site. As such, it also suggests the change is unlikely to affect either enzyme activity or substrate binding. This only partially agrees with the findings of Takahashi *et al.* [4] who showed no significant difference in *V_max_* but *did* show a reduction in *K_m_* for the K189E variant, suggesting this substitution results in stronger binding of the DHT substrate (**Table 1**).

#### R258C variant

The rs28943580 SNP results in the exchange of a large positively charged residue, arginine (R), for a smaller polar residue, cysteine (C) with a thiol group. Figure S4 shows the environment around residue 258. Both R (shown in orange) and C (shown in magenta) adopt the same rotamer. The normal (WT) R258 residue exists in a heterogeneous environment comprising polar Q262 and Q287 residues and non-polar L261 and F284 residues (shown in cyan). The interaction between the R258 and the polar residues, along with water molecules (shown in blue) around the surface of the protein stabilizes this environment.

The R258C substitution (shown in magenta) breaks these stabilizing interactions and draws the residue deeper into more hydrophobic core of the protein in proximity to V283, F286, and L288 (shown in cyan), although presumably the polar Q262 residue, also being present in the local environment, maintains some stability.

Substitution of C for R results in a slight worsening in ProSA-web z-score, from −11.63 to −11.56 and also in the Verify3D score: up to 0.55 from 0.44. The MolProbity clash score rises from 6.96 to 16.29 but no new clash arises due to the substitution. The accessible surface area drops dramatically from 27.42 to 3.85. This supports the observation above that the substitution results in the sidechain being drawn further into the core of the protein.

Once again, this amino acid substitution is located at some distance from the active site and thus suggests the change is unlikely to affect either enzyme activity or substrate binding. This only partially agrees with the findings of Takahashi *et al.* [4] who showed no significant difference in *V_max_* but *did* show a reduction in *K_m_* for the R258C variant, suggesting this substitution results in stronger binding of the DHT substrate (**Table 1**).

### AKR1C2 SND variants

The other variants examined by Takahashi *et al.* [4] were determined to be SNDs. As such, the protein variants do not occur naturally. Nonetheless, homology modeling can still be used to examine the biochemical changes resulting from these engineered mutations.

#### V38I variant

The V38I variant corresponds to the rs3207898 SND. It involves the conservative exchange of a small hydrophobic residue (valine) for a slightly larger hydrophobic residue (isoleucine). Figure S5 shows the environment around residue 38. Both V (valine, shown in orange) and I (isoleucine, shown in magenta) adopt the same rotamer. Residue 38 is located away from the active site of AKR1C2 in a pre-dominantly hydrophobic environment comprising F21, T23, I42, I49, V61, and I65 (shown in cyan). The conservative nature of the substitution and its distance from the active site suggest V38I would have no effect on enzyme activity or substrate binding. These data concur with the findings of Takahashi *et al.* [4] who showed no significant difference in either *V_max_* or *K_m_* for the V38I variant (**Table 1**).

#### V38A variant

The SND rs3207901 corresponds to the V38A variant. Like V38I described above, the variant involves a conservative exchange of one hydrophobic residue (valine) for another, slightly smaller, one (alanine). Figure S6 shows again the environment around residue 38, but with the V (valine, shown in orange) and A (alanine, shown in magenta) depicted. As noted above, the environment is pre-dominantly hydrophobic. However, the mutation to a smaller residue removes the proximal relationship between residue 38 and the surrounding residues, with the exception of F21. Nonetheless, the mutation remains distant from the active site and thus suggests that V38A would also have no effect on enzyme activity or substrate binding. This only partially agrees with the findings of Takahashi *et al.* [4] who showed no significant difference in *V_max_* but *did* show a reduction in *K_m_* for the V38A variant, suggesting this substitution results in stronger binding of the DHT substrate (**Table 1**).

#### H47R variant

The H47R variant corresponds to the rs3207905 SND. It involves a conservative exchange of one large, positively charged residue (histidine) for another (arginine). Figure S7 shows the environment around residue 47. Both H (histidine, shown in orange) and R (arginine, shown in magenta) adopt the same rotamer. H47R exists in a hydrophilic environment comprising D2, Y5, and D78 (shown in cyan), along with a number of water molecules (shown in blue). H47 is hydrogen-bonded (shown in green) to one of the water molecules while R47 forms a hydrogen bond with D2. Thus, while there is some re-arrangement of interactions, the nature of environment remains similar as a result of the exchange. The conservative nature of the substitution and its distance from the active site suggest H47R would have no effect on enzyme activity or substrate binding. These data concur with the findings of Takahashi *et al.* [4] who showed no significant difference in either *V_max_* or *K_m_* for the H47R variant (**Table 1**).

#### S87C variant

The SND rs13933 corresponds to the S87C variant. This variant involves a conservative exchange of one small polar residue (serine) for another (cysteine). The environment around residue 87 is shown in Figure S8. Interestingly, S (serine, shown in orange) and C (cysteine, shown in magenta), adopt different rotamers. S87C exists in a heterogeneous environment surrounded by a mixture of the hydrophobic residues W86, F118, V120, V122, and F311, along with the positively charged H90 (shown in cyan) and two water molecules (shown in blue). The position of residue 87 is also close to the active site, with W86 also in close proximity to the substrate. Upon exchange, the pattern of hydrogen bonding (shown in green) with the adjacent water molecules changes. Nonetheless, the conservative nature of the change does not strongly alter the local environment, suggesting S87C would have no effect on enzyme activity or substrate binding. These data concur with the findings of Takahashi *et al.* [4] who showed no significant difference in either *V_max_* or *K_m_* for the S87C variant (**Table 1**).

#### H170R variant

The H170R variant corresponds to the rs10618 SND. It involves a conservative exchange of one large, positively charged residue (histidine) for another (arginine). Figure S9 shows the environment around residue 170. Both H (histidine, shown in orange) and R (arginine, shown in magenta) adopt the same rotamer. H47R exists in a heterogeneous environment, on the surface of the molecule and distant from the active site. While close to the hydrophobic F205 and the polar Y323, it is also near the negatively charged E174 (shown in cyan) and a number of water molecules (shown in blue). Both H and R form hydrogen bonds (shown in green) with the water molecules, although the arrangement is different. The conservative nature of the substitution, its location on the surface of the molecule, and its distance from the active site suggest H170R would have no effect on enzyme activity or substrate binding. These data concur with the findings of Takahashi *et al.* [4] who showed no significant difference in either *V_max_* or *K_m_* for the H47R variant (**Table 1**).

#### L172Q variant

The SND rs11474 corresponds to the L172Q variant. This variant involves exchanging a small hydrophobic residue (leucine) for a relatively small hydrophilic residue, glutamine.

**Figure 4:**
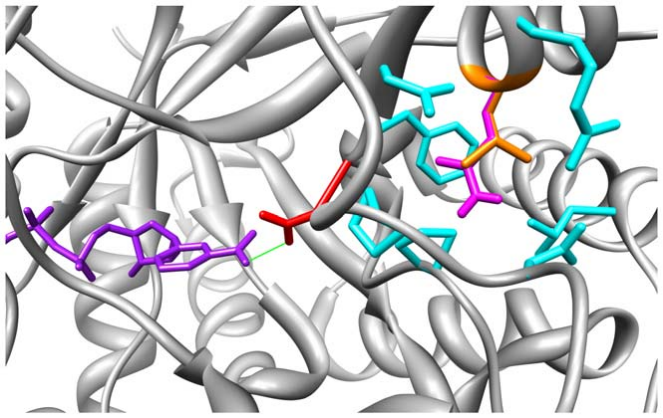
The L172Q substitution in the AKR1C2 predicted protein disrupts the non-polar environment near N167. The environment of residue 172 shows both the L and Q variants adopt the same rotamer. The variant introduces a polar residue into a largely non-polar environment close by N167, which forms a hydrogen bond with the nicotinamide ring of the cofactor.

**Figure 4** shows the environment of residue 172. Again, both L (leucine) and Q (glutamine) adopt the same rotamer. The normal (WT) L172 (shown in orange) is surrounded by two groups of residues (shown in cyan; **Figure 4**). Those closer to the surface are charged (R171) or polar (N169 and N316), with N316 and R171 sharing a hydrogen bond. In contrast, those in the interior of the molecule are non-polar (P119, L144, F168, and P318).

N167 (shown in red; **Figure 4**) is also located on the very edge of the local environment of residue 172 and closer still to the non-polar environment created by P119, F168, and P318. N167 forms a hydrogen bond (shown in green) with carboxy-amide group of the nicotinamide ring of the NADP cofactor[14] (shown in purple).

The introduction of the polar L172Q variant (shown in magenta; **Figure 4**) into the non-polar environment at the core of the protein is likely to disrupt this environment as it seeks to accommodate the new polar residue. The proximity of this non-polar environment to N167 leads to the hypothesis that it too will be affected by this adjustment, disrupting the hydrogen bond with the cofactor and hence contributing to instability in the binding of the cofactor. This disruption would be predicted to result in a drop in enzyme activity (*V_max_*) and this is confirmed by the original experimental findings of Takahashi *et al.* [4].

However, in contrast, Takahashi *et al.* [4] also show a statistically significant decrease in the *K_m_* of binding of the DHT substrate. This implies the DHT substrate is *more strongly bound* as a result of the L172Q substitution. The site of the L172Q substitution is at a distance from the binding site of the testosterone molecule in our model and thus does not provide any strong rationale for the improved binding of DHT (**Table 1**).

### The R258C AKR1C1 variant

**Figure 5:**
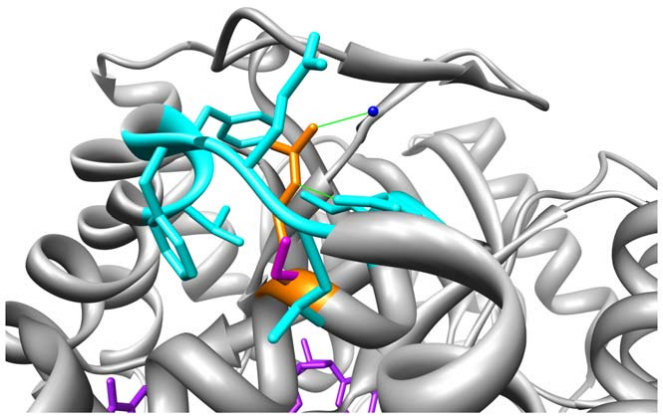
The R258C moves a polar residue into the non-polar core. The environment of residue 258 in AKR1C1 shows both the R (orange) and C (magenta) variants adopt the same rotamer. Residue 258 is located on the surface of the protein and distant from the active site. The introduction of the C residues brings the polar residue further into the non-polar core of the protein including V283, F286, and L288 (cyan).

Most of the reported SNPs in AKR1C1 are actually SNDs[8], leaving only one non-synonymous substitution, rs28943580 which results in the R258C variant which substitutes the normal arginine with cysteine, analogous to the R258C variant in AKR1C2. **Figure 5** displays the local environment of residue 258. Both the R and C residues adopt the same rotamer. While there are some slight differences in the environment of R258C in AKR1C1 and AKR1C2, the major features are similar. R258 residue exists in a heterogeneous environment comprising polar Q262 and Q287 residues and non-polar L261 and F284 residues (shown in cyan). The interaction between the R258 and the polar residues, particularly Q262, along with the water molecule (shown in blue) around the surface of the protein stabilizes this environment. The R258C substitution (shown in magenta) breaks these stabilizing interactions and draws the residue deeper into more hydrophobic core of the protein in proximity to V283, F286, and L288 (shown in cyan), although presumably the polar Q262 residue, also being present in the local environment, maintains some stability.

As for the AKR1C2 R358C substitution, in AKR1C1 the substitution results in a slight worsening in ProSA-web z-score, from −11.49 to −11.42 and also in the Verify3D score: up to 0.62 from 0.60. The MolProbity clash score rises from 7.31 to 13.55 but no new clash arises due to the substitution. The accessible surface area drops from 11.24 to 3.60, in line with the observation above that the substitution results in the sidechain being drawn further into the core of the protein.

Once again, this amino acid substitution is located at some distance from the active site and on the surface of the molecule. Thus, despite the changes noted above, the substitution does not suggest any effect on the enzyme activity of AKR1C1 or the binding of the substrate or cofactor (**Table 1**).

## Discussion

The gene products of the AKR1C1 and AKR1C2 genes catalyze important reactions in the formation and inactivation of male and female sex hormones (**Figure 1**). In their study of the enzyme kinetics of AKR1C2, Takahashi *et al.* [4] showed two non-synonymous single nucleotide changes, corresponding to the F46Y and L172Q variants, resulted in reduced enzyme activity. In this study, our modeling confirms this result, showing F46Y (the result of a SNP) and L172Q (the result of a SND) both potentially disrupt hydrogen bonding between the enzyme and the NADP cofactor (**Figure 3** and **Figure 4**). Thus, we hypothesize that the reduced enzyme activity reported by Takahashi *et al.* [4] resulting from either of these two substitutions results from a more weakly bound cofactor. This could be confirmed by measuring the *K_m_* of the cofactor in an experiment analogous to those already undertaken by Takahashi *et al.* [4] in measuring the *K_m_* of the DHT substrate. Furthermore, this hypothesis is also supported by the reduced *K_m_* of the DHT substrate in L172Q, indicating the DHT is more strongly bound, and thus not likely to be the source of the reduced enzyme activity.

The other four SNPs (V111A, K179E, K185E, and R258C) and other five SNDs (V38I, V38A, H47R, S87C, H170R) did not show reduced enzyme activity in the experimental analysis of Takahashi *et al.* [4]. Our modeling supports this finding. We have shown these substitutions to be either conservative (V111A, V38I, V38A, H47R, S87C, H170R), or to exist on the surface of the molecule and distant from the active site (K179E, K185E, and R258C), and thus they are unlikely to directly effect enzyme activity, substrate binding, or cofactor binding. We hypothesize that measurement of the *K_m_* of cofactor binding in these variants would should no significant difference to the normal (WT) enzyme.

Similarly, in modeling the R258C amino acid substitution in the predicted AKR1C1 gene product, we find the substitution occurs on the surface of the molecule, distant from the active site. We thus hypothesize that similar enzyme kinetic experiments on AKR1C1 would show that R258C does not reduce enzyme activity or significantly change either progesterone or NADP binding compared to the wild type.

One finding by Takahashi *et al.* [4] not explained by our modeling is the increased DHT binding (measured as reduced *K_m_* by Takahashi *et al.* [4]) in the V38A, L172Q, K185E, and R258C variants. It is possible that the general disruptiveness of these substitutions, in terms of broken salt bridges and hydrogen bonds, along with the drawing of polar or charged residues into the more non-polar core of the molecule (in the case of the latter three) or the removal of proximal hydrophobic interactions (in the case of V38A), may result in a general “opening up” of the core of the molecule making it easier for the DHT molecule to enter and bind in the active site. However, this is highly speculative and would need to be confirmed by further experiments. In this regard, we note that numerous crystal structures of AKR1C2 already exist (including PDB accession numbers: 2HDJ[25], 1IHI[24], 1J96[14], 1XJB[26], 2IPJ[27]). This suggests the crystallization conditions of AKR1C2 are well known, making it feasible that crystal structures of the variant proteins could be determined, potentially helping to explain any more substantial re-arrangements of structure that may occur as a result of these variants.

It is also worth considering the possibility that AKR1C2 may act as a homodimer. In this case, residues on the surface of the protein may be distant from the active site, but involved in the stabilization of the homodimer. Thus, changes to these residues may affect the function of the enzyme through destablization of the biological unit. The PDB records of the five crystal structures for AKR1C2 give mixed information regarding the nature of the biological unit, with some indicating a dimer and some a monomer. Furthermore, there is no mention of the biological unit in the research papers associated with these database entries. Nor could we find reference to the nature of the biological unit in a literature search. Using ProtCID[28], we identified that the interface in the asymmetric unit of 1J96 is shared by a 40% homologue: 2,5-diketo-D-gluconic acids reductase A from *Corynebacterium sp.* This provides some evidence to support the existence of a homodimer. Cross-referencing the list of SNP and SND variants against the residues near the interface, as provided by ProtCID, showed that only the R258C SNP and the H47R SND were near the interface, although still not making contact with residues in the other domain. Thus, even if the homodimer were confirmed, none of the variant residues considered would appear to mediate their effect on the biochemistry of the enzyme through destabilization of the dimer.

Finally, we also note the complexity of the various kinetic parameters in an ordered bi-bi reaction, such as the one catalyzed by AKR1C2. Jin and Penning[29] showed the rate determining step of the reaction catalyzed by AKR1C2 depends on both the enzyme and the substrate, with several steps, including cofactor release and steroid release contributing to the rate of reaction. Our results are compatible with this finding. In particular, we suggest the reduced enzyme activity of the F46Y and L172Q variants may be mediated through destabilization of the cofactor. Thus, the cofactor position may be altered from the ideal found in the wild type, slowing the rate of reaction. However, the complexity of the reaction dynamics also raises alternative possibilities, such as the potential for a destabilized cofactor to be released faster and thus increase the reaction rate. Our results suggest one possible mechanism and thus provide a basis for the development of further experiments to determine the contribution of these structural changes to the biochemistry of AKR1C2.

The careful and thorough experiments of Takahashi *et al.* [4] help to further highlight the potential issues that may arise from the contamination of SNP databases with single nucleotide differences (SNDs)[8]. Some implications of SNDs and other database errors have been discussed by Day[9]. In this case, eleven variants of AKR1C2 were chosen for experimental analysis on the basis of their listing in the widely used dbSNP database[7]. Expression constructs were created and rigorous kinetic experiments conducted for each variant: presumably at a considerable cost in terms of research time and consumables. Yet, reference to our SND data shows six of these variants (rs3207901, rs3207898, rs3207905, rs13933, rs10618, rs11474) to be SNDs rather than SNPs.

This in no way diminishes the work of Takahashi *et al.* [4] completed prior to the publication of our SND analysis[8]. The biochemical analysis of all the variants tested still stands and these effects can be further explored with homology modeling as we have shown. However, the significance of the findings in relation to the SND variants is reduced since these particular variants have not yet been established as natural genetic variants. For example, V38A and L172Q were shown to have a significant effect on DHT binding, but this is not as significant as the findings in relation to F46Y and the other four variants derived from SNPs, as V38A and L172Q have not yet been established to occur naturally.

The AKRIC1 and AKR1C2 genes are involved in sex steroid metabolism, metabolizing progesterone and 5α-dihydrotestosterone respectively (**Figure 1**). As mentioned previously, AKR1C1 has a role in controlling plasma progesterone levels in pregnancy [3] while AKR1C2 has been linked to prostate cancer [5], [6]. Thus, our comparative modeling and analysis of the effect of SNPs may provide useful functional insights into the role of sex steroid metabolism in pregnancy, prostate cancer, and other disorders, particularly where SNP and haplotype analysis is used.

Due to the potential for considerable time and money to be invested in studies of supposed SNPs that are actually SNDs, it important that further efforts are made to clean and curate our online SNP repositories to remove, or at least flag as “suspect”, these SNDs. Pending these changes, researchers should carefully and thoroughly explore the SNPs they choose for use in their analysis [8], [9].

## Supporting Information

Figure S1 **V111A is a conservative mutation.** The environment of residue 111 shows both the V (orange) and A (magenta) variants. The introduction of the A residue is a conservative mutation, distant from the active site, and located on the periphery of the protein.
(TIF)

Figure S2 **K179E breaks a surface salt-bridge.** The environment of residue 179 shows both the K (orange) and E (magenta) variants adopt the same rotamer. Residue 179 is located on the surface of the protein and distant from the active site. The introduction of the E residue breaks a salt bridge with nearby E149 and E152 (cyan) and brings the charge further into the non-polar core of the protein including W148, I176, and L182 (cyan).
(TIF)

Figure S3 **K185E moves a charged residue into the non-polar core.** The environment of residue 185 shows both the K (orange) and E (magenta) variants adopt the same rotamer. Residue 185 is located on the surface of the protein and distant from the active site. The introduction of the E residue brings the charge further into the non-polar core of the protein including L177 and L182 (cyan).
(TIF)

Figure S4 **R258C moves a polar residue into the non-polar core.** The environment of residue 258 shows both the R (orange) and C (magenta) variants adopt the same rotamer. Residue 258 is located on the surface of the protein and distant from the active site. The introduction of the C residue brings the polar residue further into the non-polar core of the protein including V283, F286, and L288 (cyan).
(TIF)

Figure S5 **V38I is a conservative mutation.** The environment of residue 38 shows both the V (orange) and I (magenta) variants adopt the same rotamer. The introduction of the I residue is a conservative mutation distant from the active site.
(TIF)

Figure S6 **V38A disrupts the local hydrophobic environment.** The environment of residue 38 shows both the V (orange) and A (magenta) residues. The introduction of the A residue, while conservative, does remove many proximal interactions with other hydrophobic residues.
(TIF)

Figure S7 **H47R is a conservative mutation.** The environment of residue 47 shows both the H (orange) and R (magenta) variants adopt the same rotamer. The introduction of the R residue is a conservative mutation distant from the active site.
(TIF)

Figure S8 **S87C is a conservative mutation.** The environment of residue 87 shows S (orange) and C (magenta) adopt a different rotamer. Despite this change, and close proximity to the active site, the introduction of the C residue is a conservative mutation with little effect on the local environment.
(TIF)

Figure S9 **H170R is a conservative mutation.** The environment of residue 170 shows both the H (orange) and R (magenta) variants adopt the same rotamer. The introduction of the R residue is a conservative mutation distant from the active site and located on the surface of the protein.
(TIF)
