# Supplementary figures and images for: Modeling Single Nucleotide Polymorphisms in the Human AKR1C1 and AKR1C2 Genes: Implications for Functional and Genotyping Analyses
Source: PLoS One. 2010 Dec 31;5(12):e15604. doi: 10.1371/journal.pone.0015604 (PMC3013106; doi:10.1371/journal.pone.0015604)

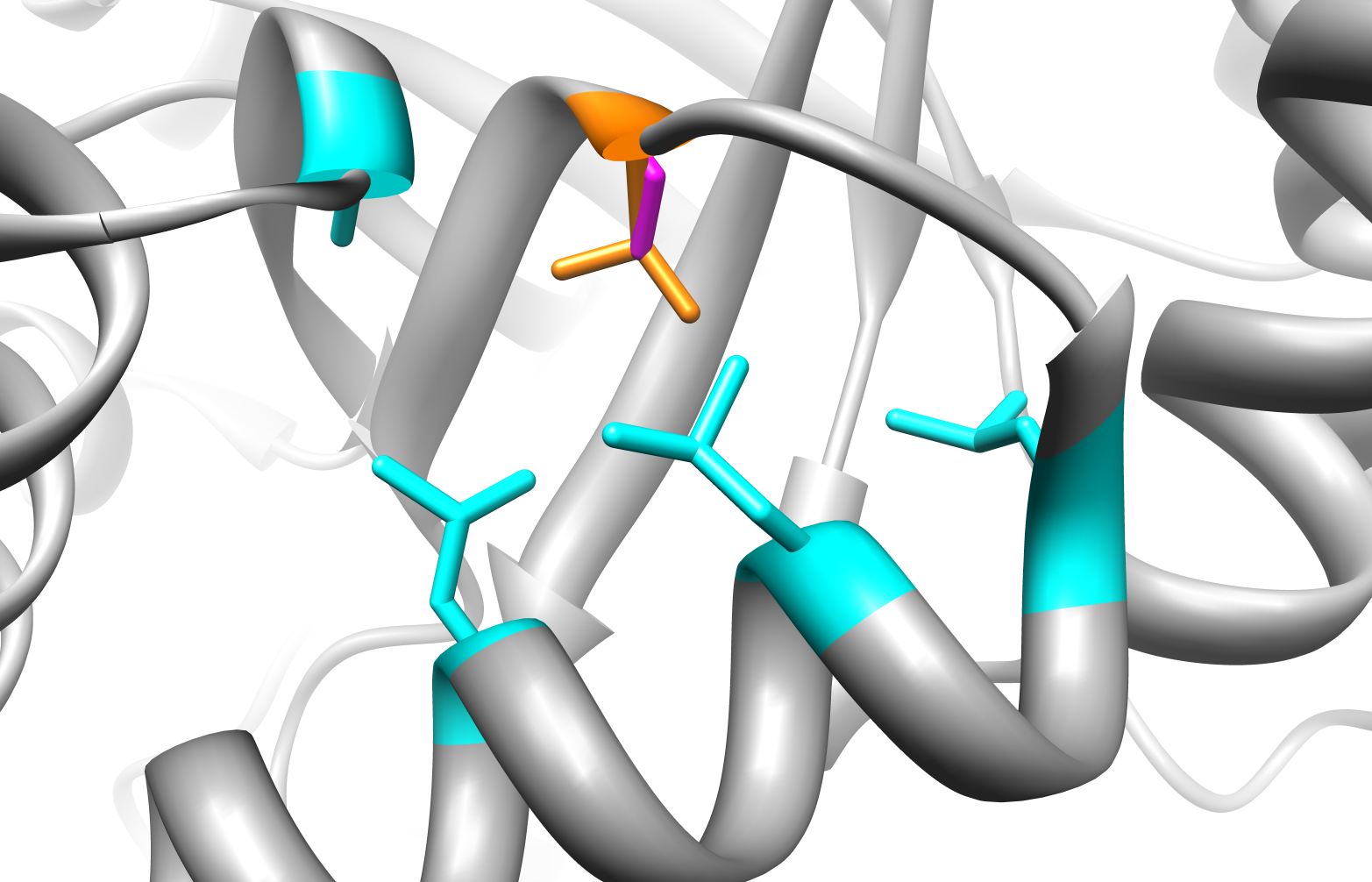

Supplement: Figure S1 — V111A is a conservative mutation. The environment of residue 111 shows both the V (orange) and A (magenta) variants. The introduction of the A residue is a conservative mutation, distant from the active site, and located on the periphery of the protein. (TIF) [file pone.0015604.s001.tif]

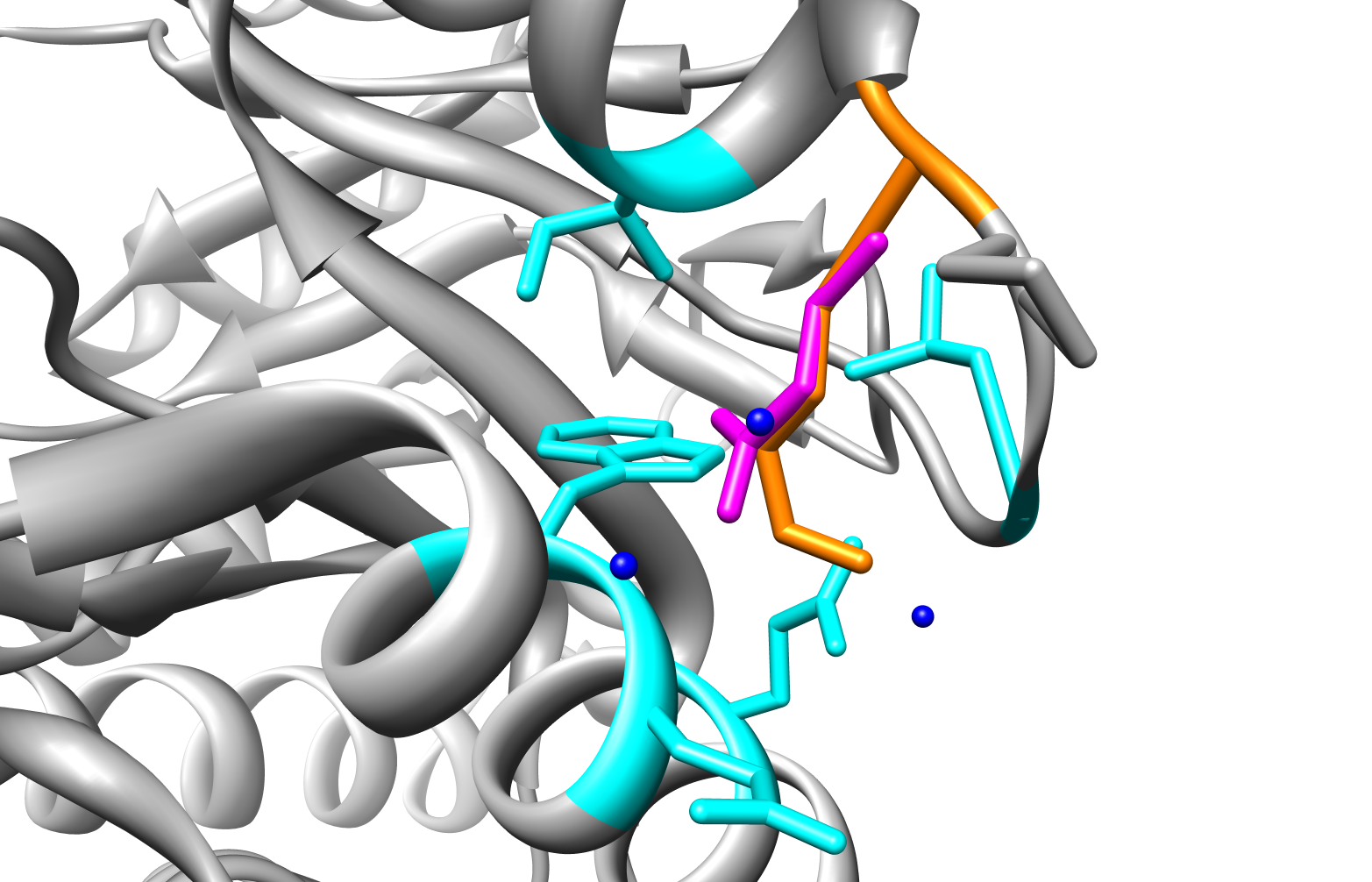

Supplement: Figure S2 — K179E breaks a surface salt-bridge. The environment of residue 179 shows both the K (orange) and E (magenta) variants adopt the same rotamer. Residue 179 is located on the surface of the protein and distant from the active site. The introduction of the E residue breaks a salt bridge with nearby E149 and E152 (cyan) and brings the charge further into the non-polar core of the protein including W148, I176, and L182 (cyan). (TIF) [file pone.0015604.s002.tif]

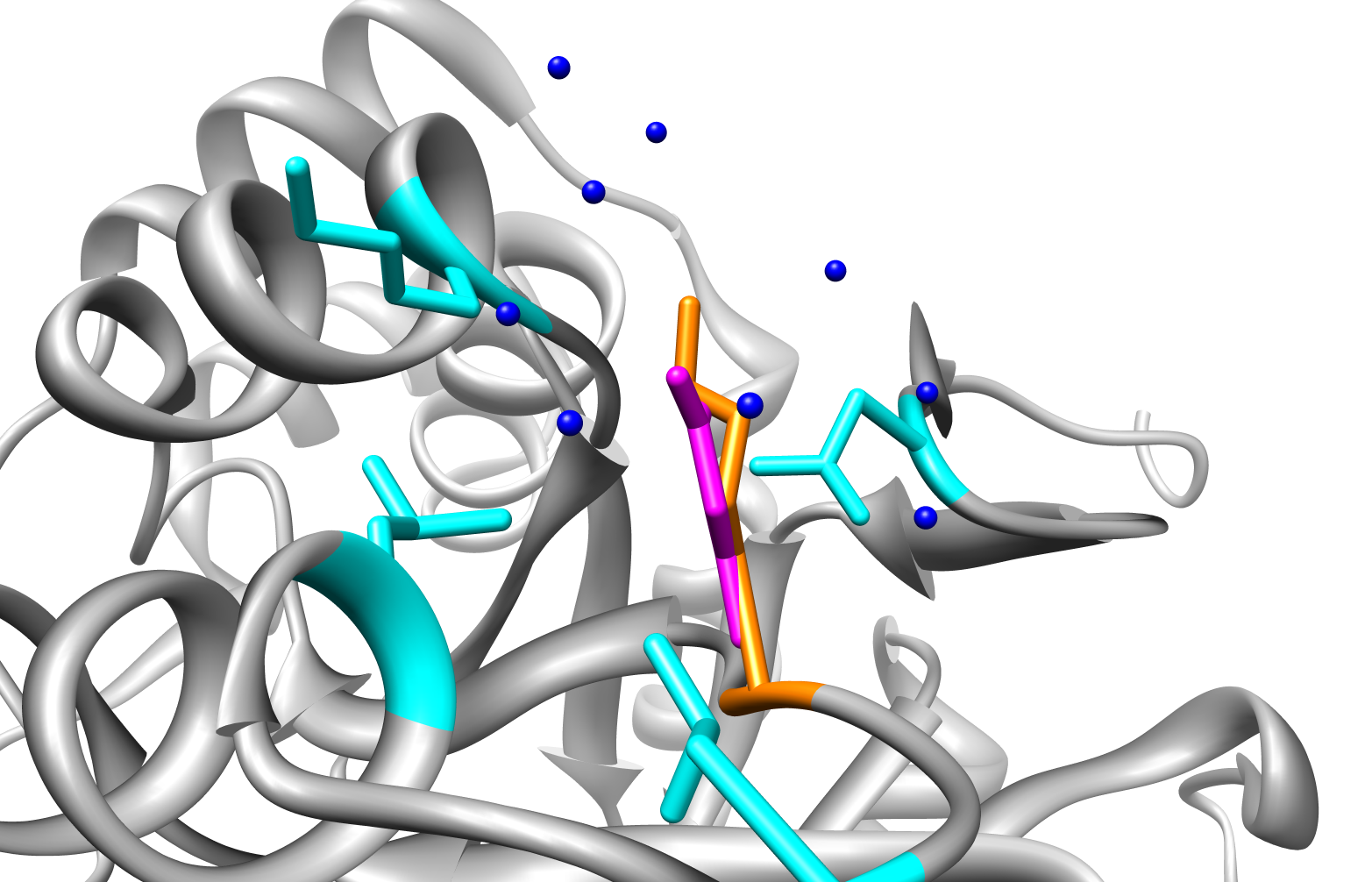

Supplement: Figure S3 — K185E moves a charged residue into the non-polar core. The environment of residue 185 shows both the K (orange) and E (magenta) variants adopt the same rotamer. Residue 185 is located on the surface of the protein and distant from the active site. The introduction of the E residue brings the charge further into the non-polar core of the protein including L177 and L182 (cyan). (TIF) [file pone.0015604.s003.tif]

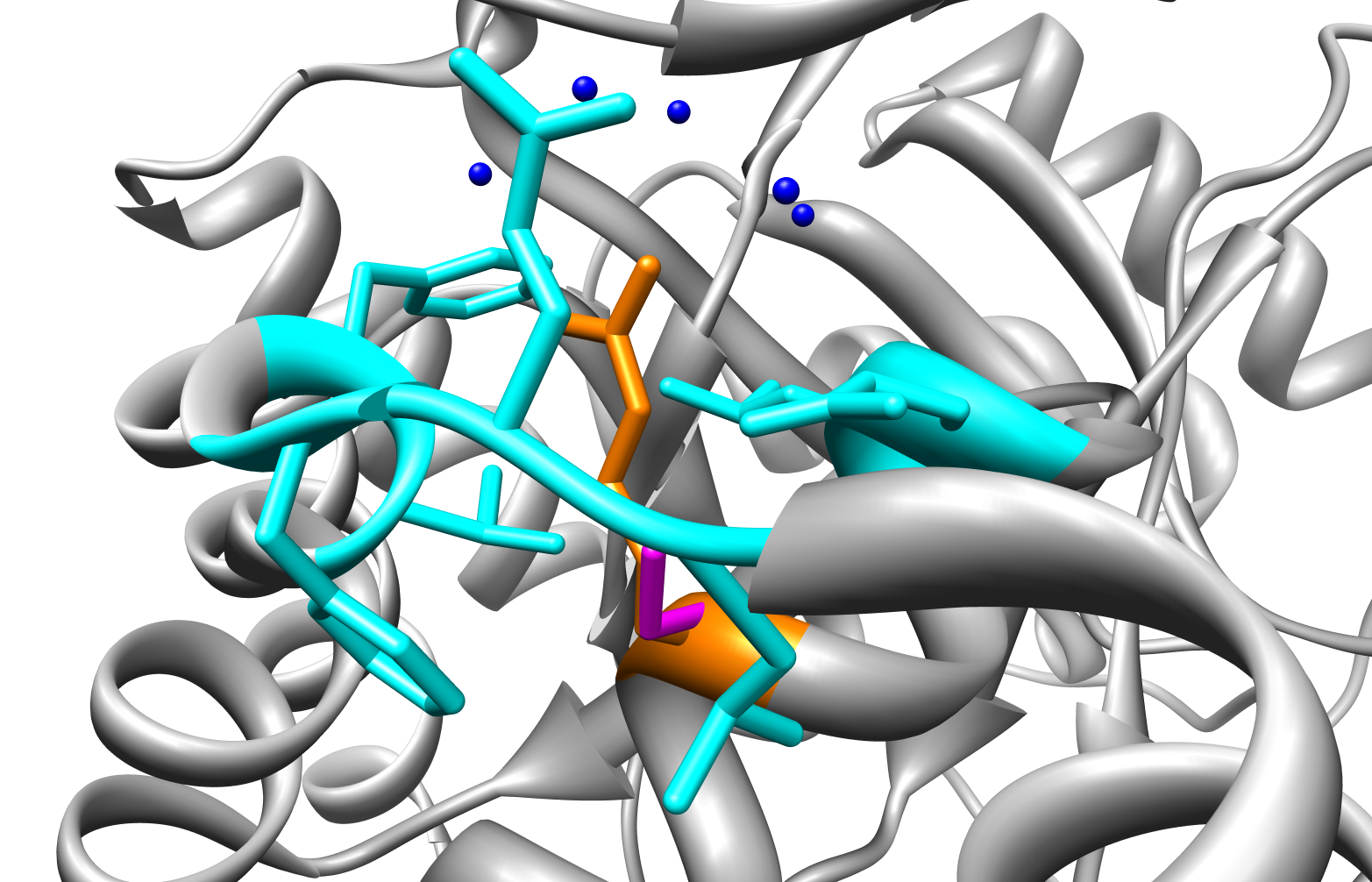

Supplement: Figure S4 — R258C moves a polar residue into the non-polar core. The environment of residue 258 shows both the R (orange) and C (magenta) variants adopt the same rotamer. Residue 258 is located on the surface of the protein and distant from the active site. The introduction of the C residue brings the polar residue further into the non-polar core of the protein including V283, F286, and L288 (cyan). (TIF) [file pone.0015604.s004.tif]

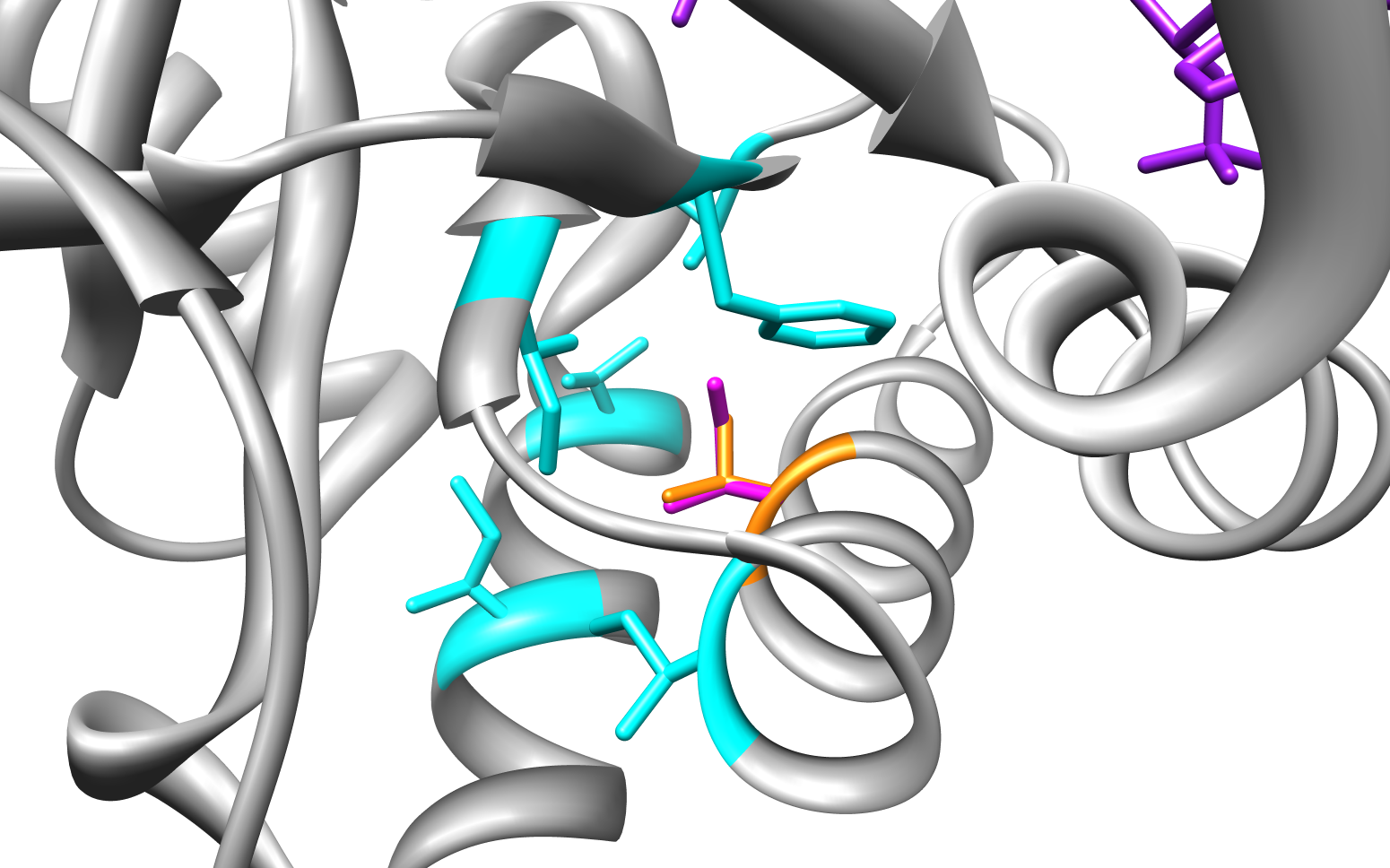

Supplement: Figure S5 — V38I is a conservative mutation. The environment of residue 38 shows both the V (orange) and I (magenta) variants adopt the same rotamer. The introduction of the I residue is a conservative mutation distant from the active site. (TIF) [file pone.0015604.s005.tif]

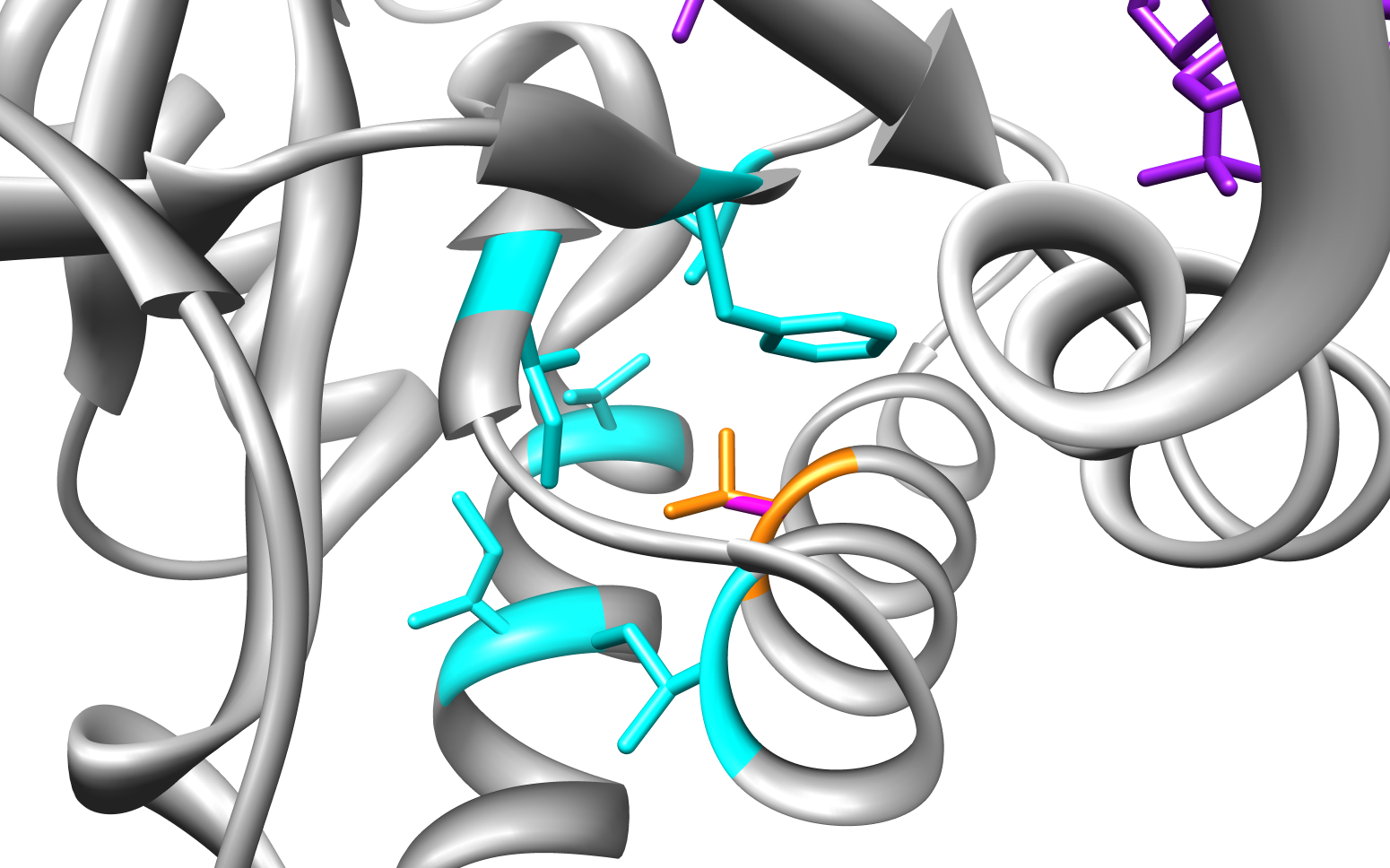

Supplement: Figure S6 — V38A disrupts the local hydrophobic environment. The environment of residue 38 shows both the V (orange) and A (magenta) residues. The introduction of the A residue, while conservative, does remove many proximal interactions with other hydrophobic residues. (TIF) [file pone.0015604.s006.tif]

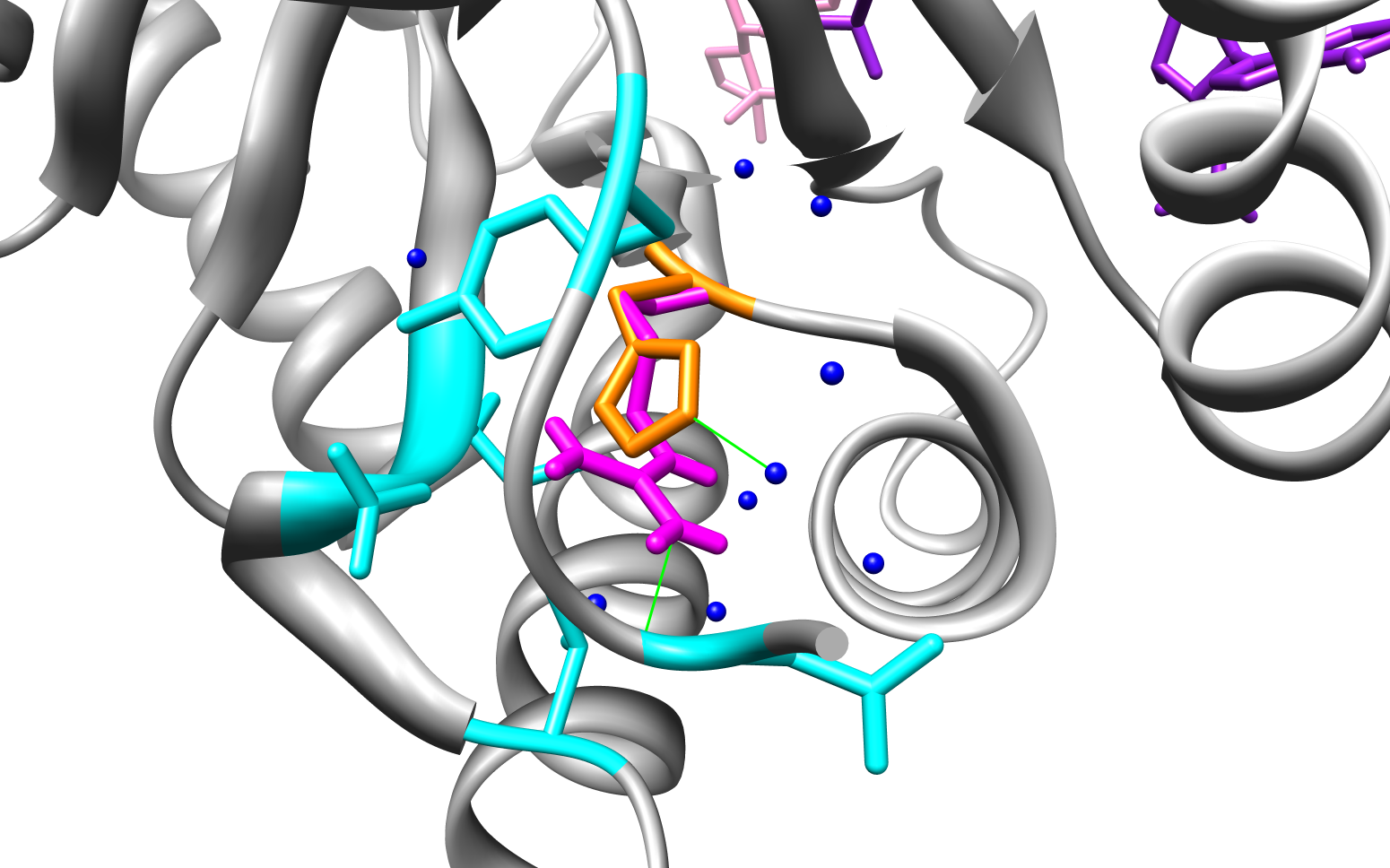

Supplement: Figure S7 — H47R is a conservative mutation. The environment of residue 47 shows both the H (orange) and R (magenta) variants adopt the same rotamer. The introduction of the R residue is a conservative mutation distant from the active site. (TIF) [file pone.0015604.s007.tif]

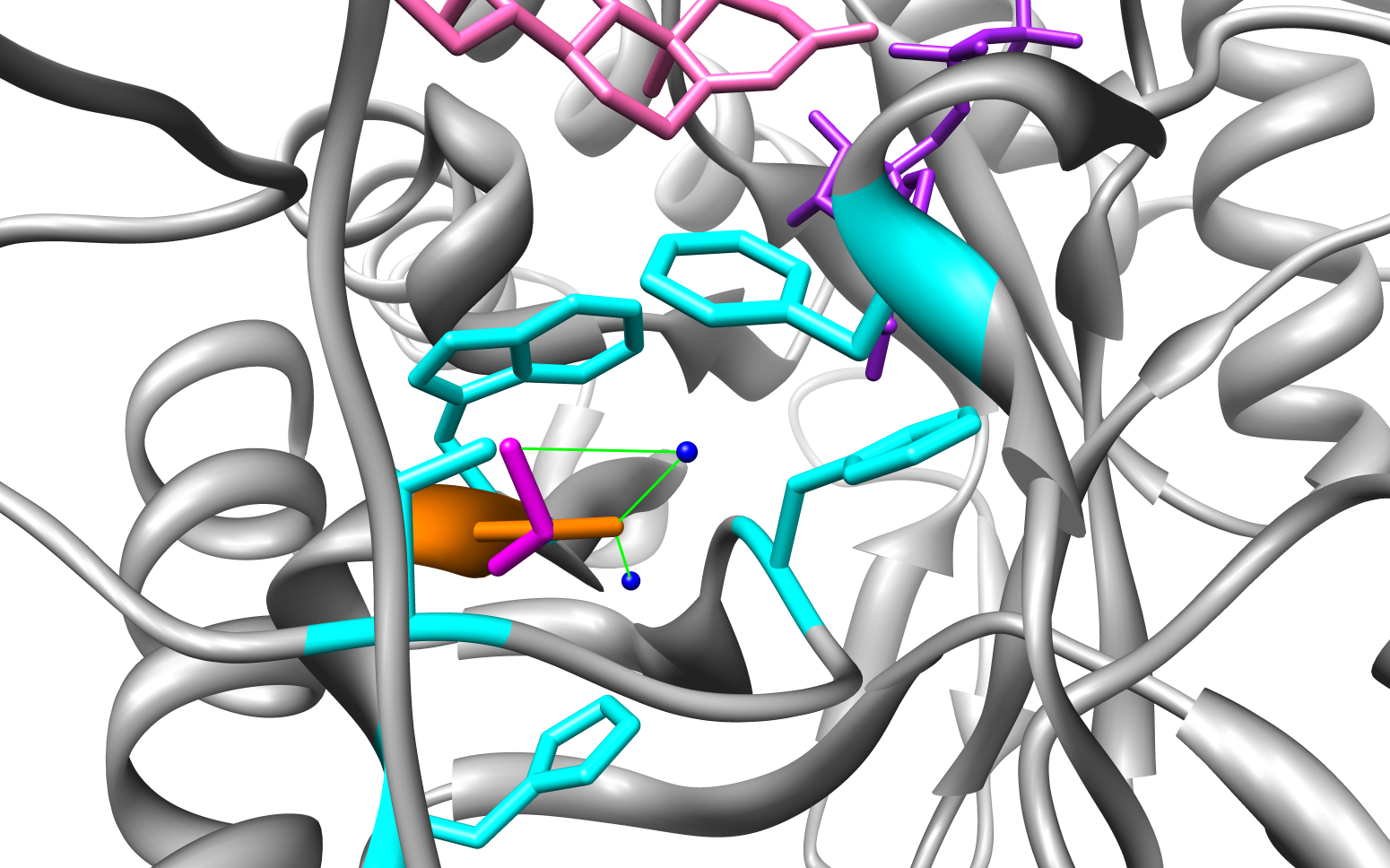

Supplement: Figure S8 — S87C is a conservative mutation. The environment of residue 87 shows S (orange) and C (magenta) adopt a different rotamer. Despite this change, and close proximity to the active site, the introduction of the C residue is a conservative mutation with little effect on the local environment. (TIF) [file pone.0015604.s008.tif]

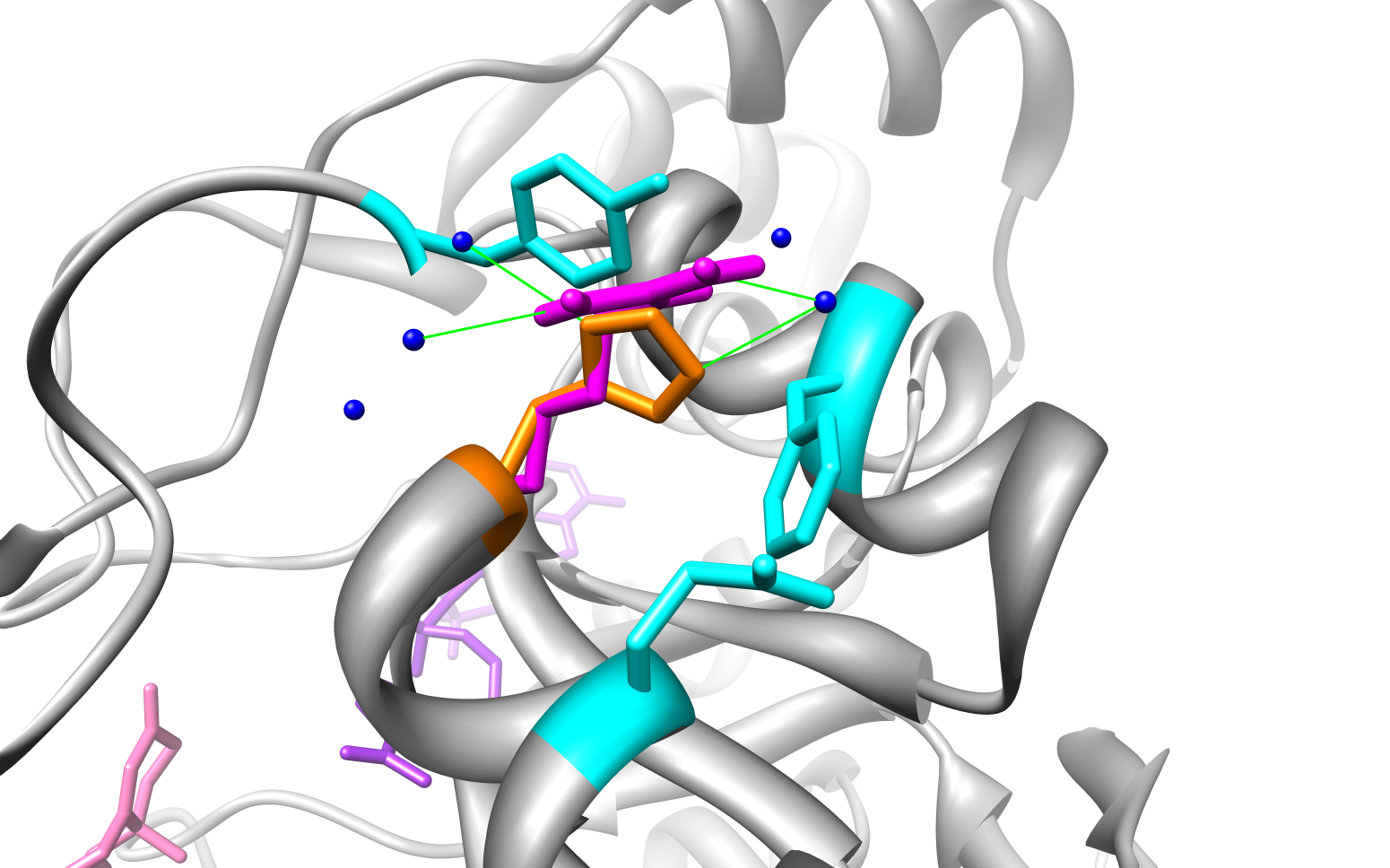

Supplement: Figure S9 — H170R is a conservative mutation. The environment of residue 170 shows both the H (orange) and R (magenta) variants adopt the same rotamer. The introduction of the R residue is a conservative mutation distant from the active site and located on the surface of the protein. (TIF) [file pone.0015604.s009.tif]
